# Fracture and Crack Behavior of Weakened Incisors Restored With Fiber Posts, Polyethylene Reinforcement, or 3D-Printed Endocrowns

**DOI:** 10.7759/cureus.92618

**Published:** 2025-09-18

**Authors:** Diana Codas-Duarte, Laís L Pelozo, Jardel F Mazzi-Chaves, Fabiane C Lopes-Olhê, Manoel D Sousa-Neto, Aline E Souza-Gabriel

**Affiliations:** 1 Department of Restorative Dentistry, Dental School of Ribeirão Preto, University of São Paulo, Ribeirão Preto, BRA

**Keywords:** cad/cam, endocrown, endodontically treated teeth, fracture resistance, ribbond

## Abstract

Background

To evaluate the biomechanical performance of weakened maxillary incisors restored with glass fiber posts (GFPs), polyethylene fiber reinforcement, or CAD/CAM (computer-aided design/computer-aided manufacturing) endocrowns (ECs), focusing on fracture resistance, failure mode, and crack formation after thermomechanical aging.

Methods

Fifty maxillary central incisors were allocated into five groups (n = 10): sound tooth (ST, control); glass fiber post (GFP) + CAD/CAM crown; polyethylene fiber in a "U" pattern (PFU) + CAD/CAM crown; polyethylene fiber in a "C" pattern (PFC) + CAD/CAM crown; and CAD/CAM EC. All experimental groups underwent root canal treatment and weakening. Specimens were subjected to 1.4 million thermomechanical cycles and loaded to fracture. Fracture mode was examined under stereomicroscopy, and cracks were analyzed via micro-CT. Fracture resistance (N) was analyzed with ANOVA and Tukey's HSD (α = 0.05), while failure and crack types were compared using Kruskal-Wallis tests.

Results

ST had the highest fracture resistance (990.8 ± 107.8 N), followed by PFC (800.5 ± 139.1 N). PFU (649.4 ± 146.8 N) and GFP (616.6 ± 122.3 N) showed intermediate values; EC had the lowest (461.1 ± 146.3 N). Most failures were favorable, but 30% of EC samples failed catastrophically. PFC and PFU preserved more crack-free roots (70% and 60%), while GFP exhibited multiple vertical cracks.

Conclusion

Polyethylene fiber reinforcement, regardless of fiber orientation, significantly improves fracture resistance and reduces root cracking in endodontically treated incisors. This technique may provide a reliable alternative to fiber posts and CAD/CAM ECs in the restorative dentistry of anterior teeth.

## Introduction

The restoration of weakened anterior teeth is a major challenge in restorative dentistry because of the high incidence of oblique forces and their aesthetic and psychological impact on patients [1]. The amount of remaining tooth structure directly influences treatment longevity and resistance [2,3], making it a key factor in selecting restorative materials and protocols. Thus, the biomechanical performance of anterior teeth depends on proper restoration [4].

Glass fiber posts (GFPs) are frequently used to restore weakened endodontically treated teeth [2,3]. Composed of glass fibers embedded in a resin matrix, they exhibit a modulus of elasticity similar to that of dentin and promote a more favorable force distribution along the root [2,3]. However, fiber posts present disadvantages such as risks of deviation or perforation, additional wear of canal walls, and the potential to induce root cracks due to undue forces [5]. Furthermore, light-curing is often inadequate in the middle and apical thirds of the canal when fiber posts are luted [6].

Endocrowns fabricated by chair-side CAD/CAM (computer-aided design/computer-aided manufacturing) have emerged as an alternative for restoring teeth with extensive tissue loss [7]. This monobloc design combines pulp chamber anchorage and the anatomical crown, requiring less root preparation than fiber posts and relying on adhesive bonding for retention [8,9]. While endocrown studies commonly focus on molars [8,10], reports on their application in anterior teeth are limited [11,12]. Moreover, crown debonding remains a concern in endocrown restorations [9].

Biomimetic dentistry offers a novel restorative philosophy aimed at preserving residual tooth structure by using biocompatible materials that mimic natural tissues, thus reducing catastrophic failures and retreatment cycles [13,14]. Within this context, biobased reinforcement with polyethylene fibers is recommended during the restoration of endodontically treated teeth.

Polyethylene fiber (Ribbond®, Ribbond, Inc., Seattle, WA, USA) allows close adaptation to tooth contours [15,16] and causes less dentinal wear compared to posts [15]. It enhances elasticity and flexural strength and reduces polymerization shrinkage in composite restorations [15]. The reinforcing effectiveness of polyethylene fibers depends on factors such as fiber size and orientation on the pulpal floor relative to applied load [16,17]. Most previous studies have evaluated polyethylene fibers on posterior teeth [15-18], demonstrating improved flexural and fracture strength of composite restorations. However, data regarding their use in anterior teeth remains limited. Only a few in vitro studies tested Ribbond® on incisors [19], and one in vivo study reported successful outcomes [20].

Given its potential as a core reinforcement material, Ribbond® may be a viable alternative to GFPs and endocrowns [21]. However, robust comparative data on its biomechanical performance in anterior teeth are lacking, where aesthetic demands and fragility create unique challenges. This study evaluated the effect of polyethylene fiber reinforcement on fracture strength and crack formation in maxillary central incisors. The null hypotheses were that GFPs, Ribbond® (circular or "U" patterns), and endocrowns would not influence fracture resistance or crack development in compromised teeth.

This article was previously posted to the University of São Paulo institutional repository as part of the Master's dissertation of Diana Codas-Duarte, submitted in November 2024.

## Materials and methods

Ethical aspects and sample size calculation

The study was approved by the local Research Ethics Committee of the School of Dentistry of Ribeirão Preto, University of São Paulo, Brazil (approval number: 69092223.8.0000.5419). A pilot study with n = 3 was done for the fracture resistance test to estimate the number of specimens required to detect a difference among at least one experimental group. Power analysis was performed using G*Power software (Heinrich Heine University Düsseldorf, Düsseldorf, Germany) with the following parameters: two-tailed test, α error = 0.05, power (1−β) = 0.80, and effect size = 0.5. A minimum of 10 specimens was determined for each group. A total of 50 teeth were selected for this study.

Teeth selection under micro-CT

Caries-free human maxillary central incisors with fully formed apices were obtained from the local Human Teeth Biobank. The teeth were selected through pre-scanning using a micro-CT device (SkyScan 1174; Bruker microCT, Atibaia, SP, Brazil) operated at 50 kV and 276 μA, with an isotropic resolution of 23.5 μm, 360° rotation around the vertical axis, a rotation step of 0.6°, a camera exposure time of 250 ms, and frame average, with a 0.5 mm-thick aluminum filter. Two-dimensional images were generated and reconstructed using NRecon v.1.6.6.0 software (Bruker microCT, Billerica, MA, USA), with artifact reduction set at 5 (scale of 0-20), beam hardening at 35% (scale of 0-100%), image smoothing at 4 (scale of 0-10), and contrast histogram range set between 0.03 and 0.15. The inclusion criteria were single-canal teeth, absence of root curvature, or pre-existing cracks. Tooth volume was equally distributed among groups.

Root canal preparation

The crowns were removed using a cutting disc (Isomet 1000; Buehler Ltd., Lake Bluff, IL, USA) at the level of the cementoenamel junction on the proximal surface, leaving 2 mm on the buccal and palatal surfaces. Endodontic treatment was performed on 40 specimens, while an additional 10 untreated teeth served as the negative control group. The root canals were prepared up to an R50 (50./05) instrument (Reciproc; VDW GmbH, Meerbusch, NRW, Germany), irrigated with 2.5% sodium hypochlorite, and dried with absorbent paper cones (Dentsply Maillefer, Ballaigues, Switzerland). Before obturation with the lateral condensation technique using a size 50 gutta-percha cone and AH Plus (Dentsply Sirona, Bensheim, Germany), the canals were irrigated with 5 mL of 17% EDTA (Sigma Aldrich, St. Louis, MO, USA) for one minute to remove the smear layer, followed by copious irrigation with saline solution. The coronal excess of the gutta-percha was removed using a heated condenser, followed by thorough cleaning and drying of the access cavities to ensure optimal sealing conditions.

Tooth weakening and group allocation

The cervical gutta-percha was removed using a heated condenser (Golgran, São Paulo, SP, Brazil), and the samples were weakened with a 3131 bur (Jota, Barcelona, CT, Spain), which was introduced in the canal until the active part reached the same level as the dentin. The teeth were divided into five groups (n = 10): Sound tooth (ST, control); translucent GFP (Exacto Angelus, Londrina, PR, Brazil) + 3D-printed resin crown; polyethylene fiber in "U" pattern (PFU; Ribbond® polyethylene fibers, Seattle, WA, USA) + 3D-printed resin crown; polyethylene fiber in "C" pattern (PFC; Ribbond® polyethylene fibers) + 3D-printed resin crown; 3D-printed endocrown (EC; priZma 3D BioCrown, Makertech Lab, São Paulo, SP, Brazil).

For the GFP group, the post space was prepared using a #2 low-speed drill (Exacto #2; Angelus), leaving a 4-mm apical seal. The root canal was irrigated and dried with paper points. The posts were cleaned with 96% alcohol, and a silane-coupling agent (Silane; Angelus) was applied. A self-etching resin cement (RelyX U-200; 3M ESPE, Saint Paul, MN, USA) was used for cementation following the manufacturer's instructions. Excess cement was removed, and the restoration was light-cured for 60 seconds (VALO; Ultradent Products, South Jordan, UT, USA).

For the PFU group, a universal bonding agent (Scotchbond™ Universal Plus; 3M ESPE) was applied to the internal surfaces of the cavity following the manufacturer's guidelines and light-cured for 10 seconds using an intensity of 1000 mW/cm² (Valo; Ultradent Products). The cavities were restored with a 10 x 3 mm polyethylene Ribbond® fiber. The polyethylene fiber was embedded into an unfilled bonding adhesive (Adper™ Scotchbond™ Multipurpose; 3M ESPE) and then pressed into the uncured filled composite (Filtek™ Z250 XT; 3M ESPE). Next, it was transferred to a dry place on a mixing pad, and the excess resin was removed by sliding a thin-bladed spatula under it. The fiber was placed and pressed as closely as possible against the tooth surfaces in a "U" pattern, going from the palatal wall to the pulpal floor until reaching the buccal wall. The fiber stopped approximately 1 mm below the cavosurface margin. Cure-light was applied for 60 seconds (Valo; Ultradent Products). The exposed portion of the fiber was coated with a low-viscosity composite resin (Filtek™ Flow; 3M ESPE).

For the PFC group, the restoration was performed as described for the PFU group, except that the polyethylene fiber was placed in a circular pattern without contacting the pulpal floor, extending through the mesial, lingual, distal, and buccal surfaces.

Then, all the cavities were restored with bulk-fill composite resin (Filtek™ Bulk Fill One Restorative; 3M ESPE) in a single 5 mm increment, followed by 60 seconds of light-curing (VALO; Ultradent Products). The core preparation for FPU and PFC crowns was carried out using diamond burs #2135 and #3118 (Jota) at high speed under abundant water cooling. The final preparation featured a rounded shoulder measuring 1.5 mm in width and 10 mm in height, with a 2 mm ferrule.

For the EC group, standardized dental preparations were performed using #2135 diamond burs. The final preparation resulted in a rounded shoulder measuring 1.5 mm in width and 10 mm in height, with a 2 mm ferrule.

3D-printing permanent crowns

The teeth were inserted into an articulated model and scanned individually using the CAD/CAM system (PrimeScan Cerec; Sirona Dental Systems GmbH, Bensheim, Germany). Digital models were generated, and the margins of each preparation were delineated using the drawing tools in Cerec Premium software (v.4.4.3; Sirona Dental Systems). The crowns were printed using a FlashForge Hunter 3D printer (FlashForge Corporation, Jiangsu, China) with an A2 color resin (priZma 3D; BioCrown Makertech Labs) and cemented with RelyX ARC (3M ESPE) following the manufacturer's protocol. Light-curing was performed for 60 seconds (Valo; Ultradent Products).

Thermomechanical aging

Thermomechanical fatigue was performed using a device (Erios Equipamentos Técnicos e Científicos Ltda., São Carlos, SP, Brazil) at temperatures of 5°C, 37°C, and 55°C, with a load of 133 N corresponding to the bite force in the anterior teeth region of patients with normal occlusion. Each specimen was subjected to 1,400,000 chewing cycles at a frequency of 2 Hz. Each temperature was maintained in contact with the specimen for 35 seconds, and each specimen underwent a total of 5,714 temperature cycles, simulating five years of degradation [22].

Fracture resistance test and fractography analysis

A compressive load was applied at a 45-degree oblique angle to simulate functional masticatory forces, with a single contact on the palatal surface, using a universal testing machine (EMIC 23-5S; Instron Corporation, Norwood, MA, USA) at a crosshead speed of 0.5 mm/min until fracture. The maximum load in Newtons (N) was recorded at the point of fracture.

The fracture analysis and crack formation were performed by three trained endodontists blinded to the study objectives and methodology. Intra- and inter-examiner reliability were determined using the kappa statistic (p > 0.80). The examiners were calibrated with reference images representing different fracture modes.

The roots were analyzed under 2.5× magnification using a stereomicroscope (Zeiss), and the fractures were classified as follows: no displacement (score 1); type I: fracture/displacement of the crown (score 2); type II: fracture in the cervical third of the root (score 3); type III: fracture up to the middle third of the root (score 4); type IV: one or multiple vertical fractures beyond the middle third of the root (score 5). Types with no displacement, I, and II were considered restorable, while types IV and V were considered catastrophic fractures (scores adapted from Silva-Sousa et al., 2020) [22].

For crack evaluation, each sample was scanned using a micro-CT device (SkyScan 1174 v2; Bruker microCT) following the same scanning and reconstruction parameters described for the pre-scanning protocol. The three-dimensional analysis of crack presence and location was performed by the same evaluators using DataViewer v.1.5.1.2 software (Bruker microCT) and CTVol v.2.2.3.0 (Bruker microCT). Cracks were classified using the following scores: (1) no defect, (2) horizontal cracks in the cervical third, (3) horizontal cracks in the middle and apical thirds, (4) vertical cracks in the cervical third, (5) vertical cracks in the middle and apical thirds, and (6) multiple crack lines (scores adapted from Rathke et al., 2022) [23].

Data analysis

Statistical analysis was performed using Statistica software (version 10.0; OBS, Lagos, Nigeria). Fracture resistance data (N) followed a normal distribution (Shapiro-Wilk test, p = .2250) and were analyzed using one-way ANOVA and Tukey's HSD test for multiple comparisons. The nonparametric Kruskal-Wallis test was used to analyze fracture types and crack formation (α=.05 for all tests).

## Results

ANOVA revealed a significant difference among the groups (p < 0.0001). The Tukey-HSD test showed that the sound teeth (ST) exhibited the highest fracture resistance, followed by Ribbond® fiber in a circular pattern (PFC), which was statistically similar to Ribbond® in a "U" pattern (PFU) (p > 0.005). Intermediate values were observed for PFU and GFP, which did not differ significantly from each other (p > 0.005). The endocrown (EC) group exhibited the lowest fracture resistance (Table 1).

**Table 1:** Fracture resistance of the experimental groups *Different capital letters indicate significant differences among groups. Sound Tooth differed from all other groups; Ribbond® U showed no significant difference from Ribbond® C and Fiber Post; and Endocrown exhibited the lowest fracture resistance values, differing from all other groups.

Groups	Fracture resistance (N)*	Statistical analysis
	(Mean ± SD)	
Sound Tooth (ST)	(990.8 ± 107.8) A	One-way ANOVA/Tukey-HSD (p < 0.0001; F-value = 31.90)
Fiber Post (GFP)	(616.6 ± 122.3) C	
Ribbond® U (PFU)	(649.4 ± 146.8) BC	
Ribbond® C (PFC)	(800.5 ± 139.1) B	
Endocrown (EC)	(461.1 ± 146.3) D	

**Figure 1:**
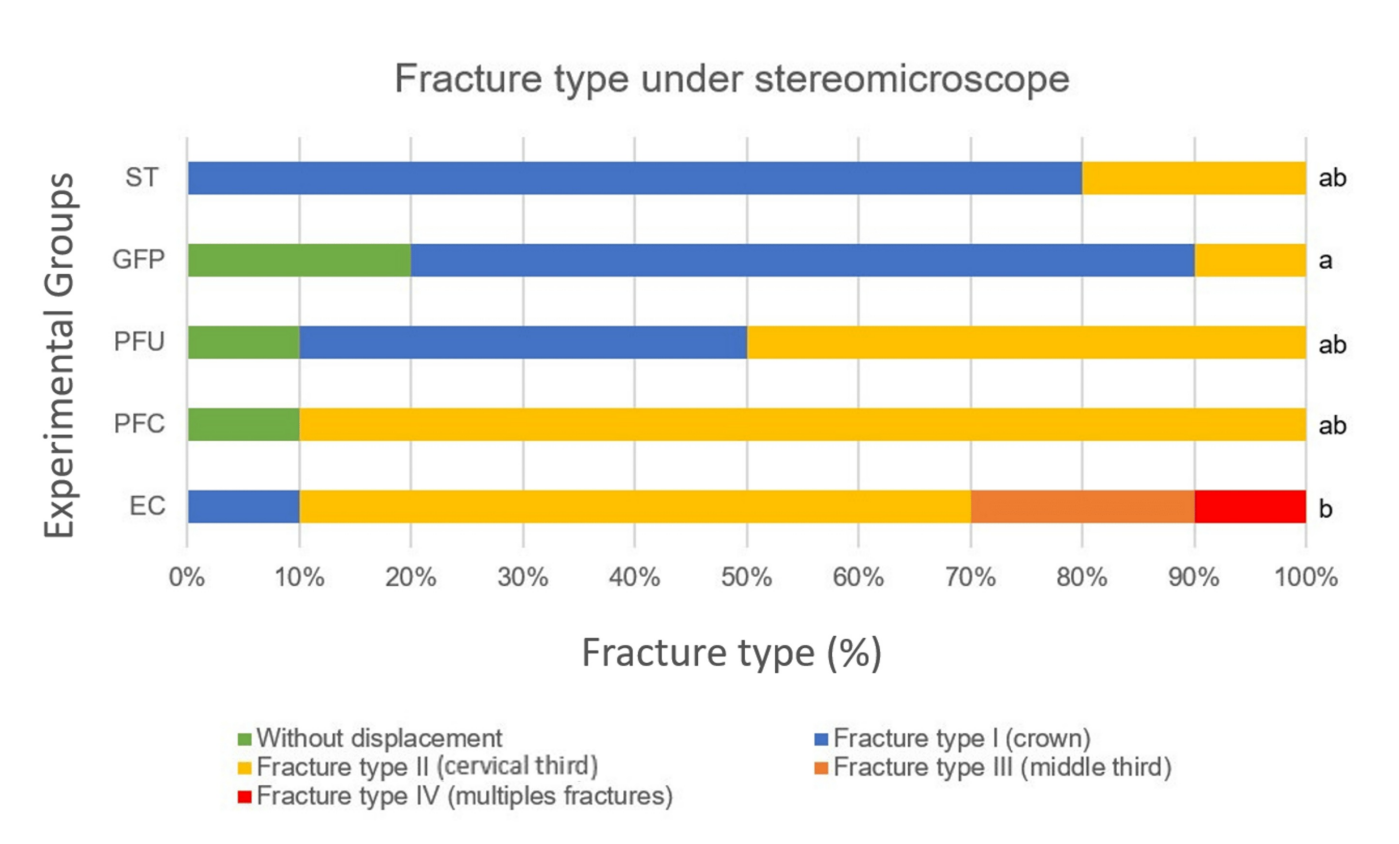
Restoration fracture types after thermomechanical cycling and fracture resistance testing. Sound Tooth (ST); Glass Fiber Post (GFP); Polyethylene Fiber in U pattern (PFU); Polyethylene Fiber in Circular pattern (PFC); and Endocrown (EC) Kruskal-Wallis multiple comparisons revealed significant differences among groups (p = 0.0003; F-value = 31.90), with GFP (a) and EC (b) differing from each other. ST, PFU, and PFC (ab) showed no significant differences from other groups.

Fracture analysis revealed that 80% of the ST presented crown fractures (Type I). Teeth restored with GFP had the highest number of samples without crown displacement, followed by the polyethylene fiber groups. In the PFU group, nearly half of the samples showed crown fractures/displacement (Type I), while in the PFC group, 90% of fractures were located in the cervical third of the root (Type II). All specimens in the EC group exhibited fractures, with 30% classified as catastrophic (Types III and IV), which differed from those in all other groups (Table 2, Figure 1).

**Table 2:** Fracture type of the experimental groups *Different lowercase letters indicate significant differences among groups. Fiber Post and Endocrown differed from each other. Sound Tooth, Ribbond® U, and Ribbond® C showed no significant differences compared with the other groups.

Groups	Fracture type under stereomicroscope*		Statistical analysis
	(Mean ± SD)	Median	
Sound Tooth (ST)	(2.0 ± 0.4)	2.0 ab	Kruskal-Wallis multiple comparisons (p = 0.0003; H-value = 25.40)
Fiber Post (GFP)	(2.0 ± 0.5)	2.0 a	
Ribbond® U (PFU)	(2.0 ± 0.6)	3.0 ab	
Ribbond® C (PFC)	(3.0 ± 0.6)	3.0 ab	
Endocrown (EC)	(3.0 ± 0.8)	3.0 b	

**Figure 2:**
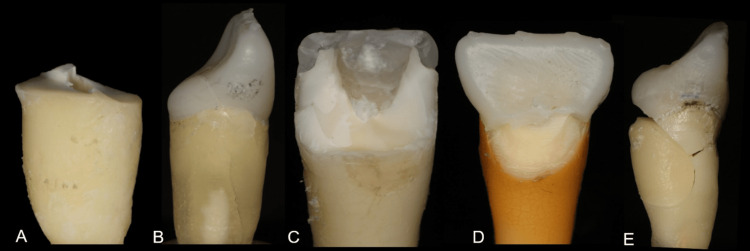
Representative photographs of the most prevalent fracture type in each experimental group (A) Sound Tooth (ST) showing the crown fracture (Type I); (B) Glass Fiber Post (GFP) represented by a sample without crown displacement; (C) Ribbond in U pattern (PFU) showing the crown fracture (Type I); (D) Ribbond in Circular pattern (PFC) with a fracture located in the cervical third of the root (Type II); (E) Endocrown (EC) demonstrating a catastrophic fracture (Type IV).

Figure 2 shows representative photographs of the most prevalent fracture types in each experimental group.

**Figure 3:**
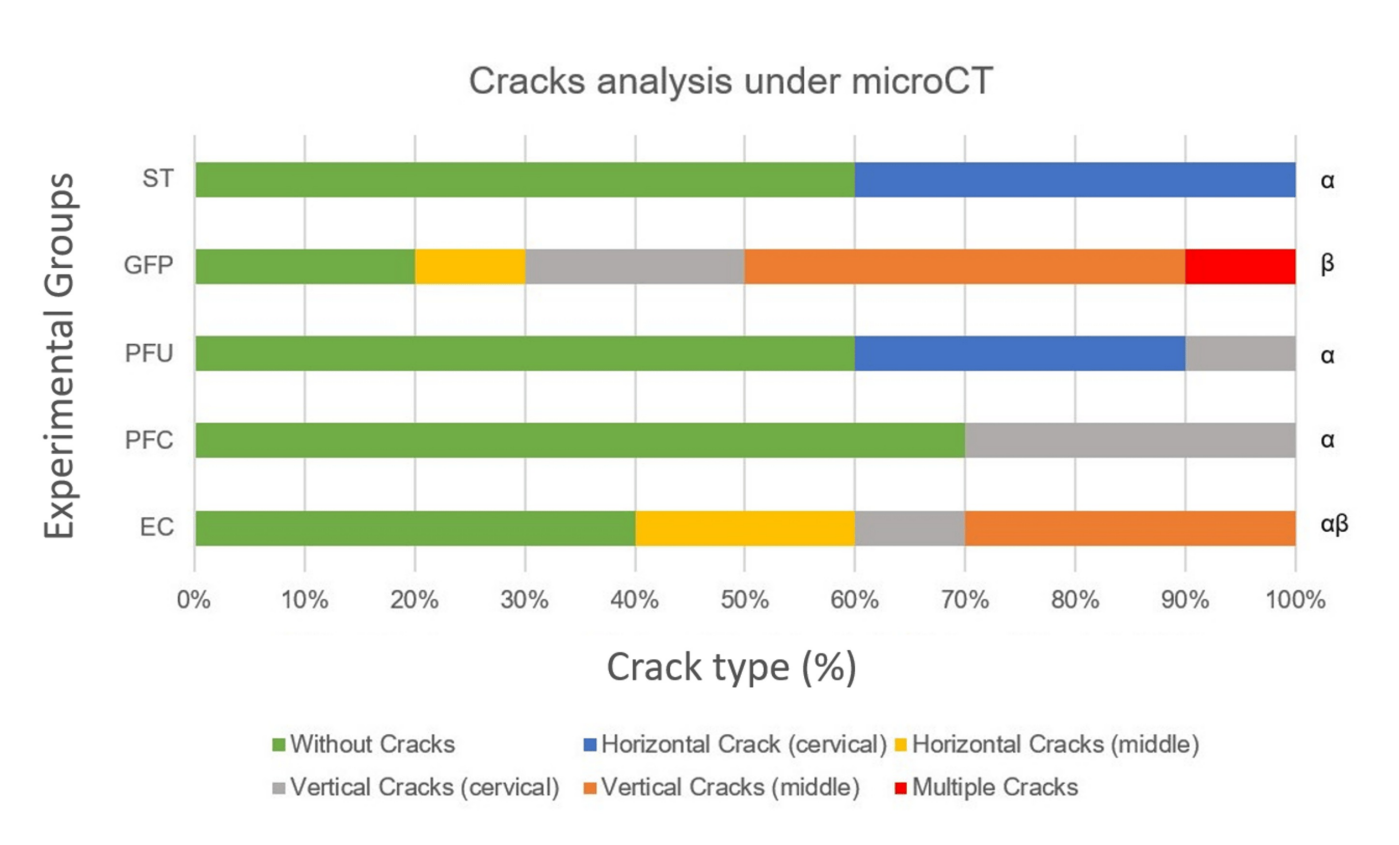
Crack analysis under micro-CT after thermomechanical cycling and fracture resistance testing. Sound Tooth (ST); Glass Fiber Post (GFP); Polyethylene Fiber in U pattern (PFU); Polyethylene Fiber in Circular pattern (PFC); and Endocrown (EC) Kruskal-Wallis multiple comparisons revealed significant differences among groups (p = 0.0008; H-value = 18.64), with GFP (β) differing from all other groups, except EC (αβ), which had no differences from any experimental group. ST, PFU, and PFC (α) showed no significant differences from each other.

Crack analysis showed that the ST had the highest prevalence of roots without cracks, though 40% presented horizontal cracks in the cervical third. The GFP group had the greatest number of samples with cracks, including 10% with multiple crack lines and 40% with vertical cracks extending to the middle and apical thirds. The PFU group showed 60% of roots without cracks and 30% with horizontal cracks in the cervical third. In the PFC group, 70% of roots were free of cracks, while the remaining samples exhibited vertical cracks in the cervical third. The EC group presented 40% of roots with vertical cracks and 20% with horizontal cracks reaching the middle and apical thirds (Table 3, Figure 3).

**Table 3:** Cracks analysis of the experimental groups *Different lowercase Greek letters indicate significant differences among groups. Sound Tooth, Ribbond® U, and Ribbond® C differed from Fiber Post. The Endocrown group showed intermediate values, not differing from the other groups.

Groups	Cracks analysis under microCT*		Statistical analysis
	(Mean ± SD)	Median	
Sound Tooth (ST)	(1.0 ± 0.5)	1.0 α	Kruskal-Wallis multiple comparisons (p = 0.0008; H-value = 18.64)
Fiber Post (GFP)	(4.0 ± 0.7)	4.5 β	
Ribbond® U (PFU)	(2.0 ± 0.9)	1.0 α	
Ribbond® C (PFC)	(2.0 ± 0.4)	1.0 α	
Endocrown (EC)	(3.0 ± 0.7)	3.0 αβ	

**Figure 4:**
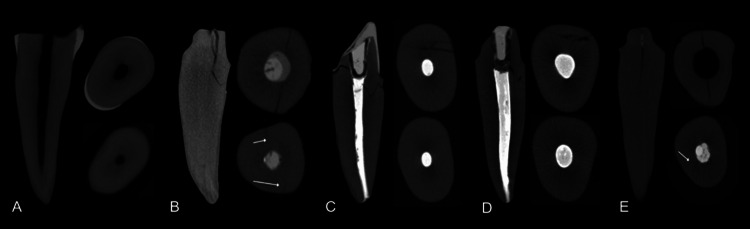
Crack analyses of teeth under micro-CT (A) Sound Tooth (ST) showing a root without a crack; (B) Glass Fiber Post (GFP) root with a vertical crack reaching the middle third of the root; (C) Ribbond in U pattern (PFU) root with a cervical horizontal crack; (D) Ribbond in Circular pattern (PFC) showing a vertical crack reaching the cervical third of the root; (E) Endocrown (EC) with a vertical crack reaching the middle third of the root. For each experimental group: left, sagittal view; right above, axial slice of cervical third; right below, axial slice of apical third.

Figure 4 illustrates the micro-CT crack analysis for all groups.

## Discussion

Structure loss in endodontically treated teeth increases the risk of root cracks and fractures [24,25]. Materials used for crown restoration should not only replace the lost enamel and dentin but also preserve the remaining healthy tissue while providing strength and rigidity to the root [23,26]. Following the correct indications and use of dentin-like CAD/CAM materials may help reduce the adverse effect of stress on the root canal.

In this study, maxillary incisors were selected due to their frequent traumatic involvement, related to their arch position and axial loading patterns [1]. Aesthetic demands challenge anterior tooth restoration, as they impact essential functions like chewing, smiling, and speech. The 1,400,000 chewing cycles and 5,714 thermocycles simulated five years of clinical thermomechanical fatigue [22].

The present study evaluated anterior teeth reinforced with polyethylene fibers and restored using resin-printed crowns reinforced with zirconia particles. Two patterns of fiber orientation ("U" or circular) were compared to ECs and GFPs to determine the most effective approach in terms of restoration stability and durability. PFU and PFC groups demonstrated higher fracture strength than the others, with no significant difference between them, supporting previous findings that fiber orientation does not affect performance [16]. Strict adherence to the adhesive protocol likely contributed to the favorable performance with Ribbond® fibers. The dentin was treated with a universal adhesive in self-etching mode, which preserves collagen and improves bonding. The polyethylene fibers were embedded in an unfilled adhesive and pressed against the composite resin, removing excess material. Such close adaptation enhances fracture resistance compared to techniques using flowable resin before fiber placement. As a result, the method promotes the formation of a strong "monobloc."

The lower fracture resistance of the fiber posts compared to polyethylene fibers may be due to the canal preparation before post cementation, which causes dentin removal along the root and further weakens the remaining structure [5]. The fracture strength results of the ST were the highest among all experimental groups, as expected. This can be explained by the intact tooth structure and the absence of dentin removal.

Conversely, ECs showed the lowest fracture resistance among all groups. This may be attributed to the extensive dentin removal required for their preparation, the absence of wall reinforcement, and the effects of axial compressive forces. Our findings corroborate previous studies that noted the critical stress concentration at the crown-tooth interface in EC restorations, which increases the vulnerability to root fracture during masticatory function [7,8].

Favorable fractures were prevalent across all groups. Groups featuring any reinforcement material (GFP, PFC, PFU) and sound tooth (ST) exhibited 100% favorable or repairable fractures. In contrast, the EC group showed a 30% rate of catastrophic fractures, corroborating previous studies [7,8]. Therefore, the indication of ECs for anterior teeth remains controversial, as the restoration of severely compromised endodontically treated anterior teeth is clinically challenging and the literature lacks consensus on the most effective materials and techniques.

The concept of bite force refers to the amount of force exerted by the masticatory muscles onto the occlusal surfaces of the teeth. In adult men experiencing stress-related factors, the maximum bite force in the molar region is typically below 350 N [27]. For anterior teeth, this force is approximately 40% of the force exerted by posterior teeth. In this study, the forces recorded to fracture the restorations exceeded these values, suggesting that restoration failure under normal conditions would be rare. Therefore, we also analyzed crack formation, as it is likely to have a more direct influence on clinical outcomes.

Crack analysis was performed using micro-CT scanning and image reconstruction, which allowed three-dimensional visualization of the roots, enabling identification of cracks on the cement surface and evaluation of crack propagation through the dentin [25].

The PFC and PFU predominantly experienced Type II fractures (fractures in the cervical third of the root) but also demonstrated a high prevalence of no crack formation in the middle and apical dentin. Besides requiring higher forces to fracture, these fiber-reinforced groups had more than half of the samples showing no crack propagation at all. These findings confirm that polyethylene fibers can arrest cracks and enhance the fracture resistance of restorations [10].

The GFP group exhibited the highest frequency of purely coronal fractures (Type I); it also resulted in vertical cracks in the cervical, middle, and apical thirds, along with multiple root fissures. This observation can be explained by the fiberglass post's ability to absorb forces and distribute them along the entire root and by the occurrence of root dentin defects following the use of various post-space preparation drills [10,28].

The propagation of vertical cracks can lead to catastrophic root fractures in the long term and is a common cause for the extraction of root-filled teeth, whereas horizontal crack propagation may be repairable [26]. These vertical and multiple cracks, present in 70% of roots restored with fiber posts, highlight the risks to long-term tooth survival. ECs exhibited favorable and catastrophic fractures, with vertical and horizontal crack propagation along the root, suggesting that the damage is primarily attributed to the root rather than the restoration itself.

Polyethylene fibers improved fracture resistance and reduced vertical crack propagation in maxillary incisors, outperforming GFPs and ECs. However, considering the inherent limitations of an *in vitro* study, the results may not fully replicate clinical conditions, including the influence of the oral environment, particular patients' occlusal forces, and long-term aging. Further clinical trials are necessary on anterior teeth with various restorative approaches to clarify the effects of polyethylene fibers, as they appear to be a biomimetic alternative technique for preserving remaining healthy tissue.

## Conclusions

Based on the findings of the present study, polyethylene fiber reinforcement significantly enhanced fracture resistance and limited root crack propagation in endodontically treated maxillary incisors, regardless of fiber orientation. GFPs exhibited the highest crown displacement without root fracture; however, they induced vertical cracks along the root with multiple fissures. In contrast, ECs presented the lowest fracture resistance and demonstrated both favorable and catastrophic fracture patterns, with vertical and horizontal cracks propagating along the root.
